# AP-1 controls the p11-dependent antidepressant response

**DOI:** 10.1038/s41380-020-0767-8

**Published:** 2020-05-21

**Authors:** Revathy U. Chottekalapanda, Salina Kalik, Jodi Gresack, Alyssa Ayala, Melanie Gao, Wei Wang, Sarah Meller, Ammar Aly, Anne Schaefer, Paul Greengard

**Affiliations:** 1 Laboratory of Molecular and Cellular Neuroscience, The Rockefeller University, 1230 York Avenue, New York, NY 10065 USA; 2 Friedman Brain Institute, Department of Neuroscience, Icahn School of Medicine at Mount Sinai, New York, NY 10029 USA

**Keywords:** Neuroscience, Biochemistry, Drug discovery, Diseases, Biomarkers

## Abstract

Selective serotonin reuptake inhibitors (SSRIs) are the most widely prescribed drugs for mood disorders. While the mechanism of SSRI action is still unknown, SSRIs are thought to exert therapeutic effects by elevating extracellular serotonin levels in the brain, and remodel the structural and functional alterations dysregulated during depression. To determine their precise mode of action, we tested whether such neuroadaptive processes are modulated by regulation of specific gene expression programs. Here we identify a transcriptional program regulated by activator protein-1 (AP-1) complex, formed by *c-Fos* and *c-Jun* that is selectively activated prior to the onset of the chronic SSRI response. The AP-1 transcriptional program modulates the expression of key neuronal remodeling genes, including *S100a10* (p11), linking neuronal plasticity to the antidepressant response. We find that AP-1 function is required for the antidepressant effect in vivo. Furthermore, we demonstrate how neurochemical pathways of BDNF and FGF2, through the MAPK, PI3K, and JNK cascades, regulate AP-1 function to mediate the beneficial effects of the antidepressant response. Here we put forth a sequential molecular network to track the antidepressant response and provide a new avenue that could be used to accelerate or potentiate antidepressant responses by triggering neuroplasticity.

## Introduction

Major depressive disorder (MDD) is a disabling psychiatric disorder with diverse etiology, and is a leading cause of mortality and morbidity worldwide [1]. The symptoms of depression are varied, and include cognitive, motivational, emotional, and physiological changes [2]. Evidence for molecular and cellular alterations in depression includes reduction in neuroplastic properties, reduced levels of neurotrophins, and decreased neurogenesis [3–5]. Clinical imaging and postmortem studies indicate functional and structural alterations in the limbic brain regions, prefrontal cortex (PFC), hippocampus, and amygdala [4, 6] in depressed patients. Many psychopharmacological agents are currently used for the treatment of depression, among which the selective serotonin reuptake inhibitors (SSRIs) are the most effective and widely prescribed [7]. However, clinical studies show that only two-thirds of patients respond to treatment, and that treatment has a delayed onset of action [8]. The therapeutic response to chronic antidepressant treatment is thought to be mediated by neuroadaptive changes in specific neuronal networks, which collectively reverses the dysregulation caused by depression [3]. There is therefore a pressing need to elucidate the identity of molecular pathways that mediate long-term treatment and antidepressant efficacy.

Gene expression regulation represents a general mechanism through which mature neuronal circuits control their physiology and behavior [9, 10]. Several transcription factors have been identified as the first responders to extracellular signals. Such factors include the immediate early genes (IEGs) such as *c-Fos, FosB, Fra1, Fra2, c-Jun, Junb, Jund, Egr1, Nr4a1*, and *Npas4*. These IEGs initiate and maintain gene expression profiles of crucial effector proteins, causing sustainable changes in the structure and function of mature synapses and thereby promoting behavioral plasticity. So far, among the IEGs, *Creb1* and *Egr1* have been previously shown to be associated with the antidepressant response [11–13]. Additionally, activation of cAMP, PKA, CAMK, and BDNF signaling molecules have been implicated in the chronic antidepressant response [14–16], likely through the regulation of physiological processes, including neuroplasticity, neuroprotection, and neurogenesis. However, how drug treatment is specifically coupled to transcription and how the signaling molecules are activated in response to chronic antidepressant administration are still unclear. Within the corticolimbic network [17], we chose to focus on the cortex, as the integral role of this brain-region in the regulation of behavior and the control of stress reactivity has been well characterized in patients and animal models [18–21]. Additionally, as the primary role of the serotonin-dependent function of the cortical circuit in the effective treatment of depression has been well established, studying the cortex would allow us to further delineate the molecular mechanisms regulating the complex response to antidepressants.

In this study, we investigated which of the IEGs are activated in the cortex by chronic treatment with an SSRI, fluoxetine, and we elucidated the target genes regulated by these factors. Furthermore, we addressed whether the IEGs and their target genes contribute directly to the behavioral response. Our findings reveal activation of a network of molecules that are sequentially linked together to provide a robust antidepressant response.

## Method details

### Treatments, transfections, and DNA constructs

*For chronic drug and JNK inhibitor treatment,* BALB/cJ mice (Jackson Laboratories) were housed two per cage and fluoxetine hydrochloride (Sigma) at a dose of (0.167 mg/mL) was administered in drinking water in 1% saccharine solution to mask the taste of the drug. Saccharine alone was given to the vehicle-treated animals. Mice were treated on average for 28 days and replaced with fresh solution every 3 days. On average, the fluoxetine-receiving mice drank approximately ∼3–4 mL a day, somewhat less compared with the control mice (that received 1% saccharine), which drank approximately ∼5–7 mL a day presumably due to the taste of the drug. The drinking volume of the fluoxetine-treated mice eventually normalized to that of the control mice. The fluoxetine-treated mice thus received 16–23 mg/kg/day of fluoxetine, an effective dose that is known to produce an antidepressant response in different strains of mice [22]. BALB/cJ mice were used as these mice are inherently anxious and show a robust antidepressant response. The S100a10-EGFP/Rpl10a ES691 mice of C57BL/6 background also produces a robust antidepressant response to fluoxetine treatment. For JNK inhibitor treatment in vivo, 16 mg/kg was injected intraperitoneally (i.p.) to the BALB/cJ mice on days 4, 6, 8, 10 of fluoxetine treatment to block JNK function to block c-Jun phosphorylation.

*For growth factor stimulation experiments* in PC12/TrkB cells and primary mixed cortical neurons, growth factors BDNF (50 ng/mL) [23, 24], FGF2 (50 ng/mL) [25], EGF (100 ng/mL) [26], IGF (100 ng/mL) [27, 28], NGF (100 ng/mL) [29], VEGF (100 ng/mL) [30], BMP4 (100 ng/mL) [31], TGFβ (100 ng/mL) [32], Bicuculline (50 μM) [33], and KCl (55 mM) [34], all from Sigma, were acutely applied onto cells. Samples were collected at 2 h to assess *c-Fos* or *c-Jun* expression, and samples were collected at 12 and 24 h for p11 expression in PC12-TrkB cells and primary mixed cortical neurons, respectively. For the BDNF- and FGF2-stimulated time course experiments, samples were collected at 2, 6, 12, 24, and 48 h after an acute application. The experiments were conducted in a 12-well plate with at least three biological replicates.

*For inhibitor experiments in primary cortical neuronal cultures*, we tested which pathways affect basal *c-Fos* and *c-Jun* transcription. We applied inhibitors to block the various molecules in the tyrosine kinase pathway. To test the BDNF- and FGF2-inducible *c-Fos* and *c-Jun* transcription, we treated cells with inhibitors 30 min before stimulation. We used inhibitor concentrations that were previously known to induce neurogenic and neurotrophic effects: TrkB (K252a, 1 μM) [35], MAP kinase kinase, MEK1 and MEK2 (U0126, 10 μM, EMD Millipore) [36], PI3K (LY294002, 50 μM) [35], PLCγ (U73122, 10 μM) [37], p38 MAPK (SB203850, 20 μM) [38], and JNK (SP600125, 20 μM) [39]. All inhibitors were bought from Sigma unless otherwise mentioned. The experiments were conducted in a 12-well plate with at least three biological replicates.

*For transfections of transcription factor small interfering RNA (siRNA*), experiments were done in PC12-TrkB cells. To identify the transcription factor regulating p11, inhibition of transcription factors were done upon treatment with two pre-validated silencer select siRNAs (Thermo Fischer Scientific) for each transcription factor according to the manufacturer’s instructions. siRNAs were transfected using Lipofectamine RNAiMAX reagent (Thermo Fischer Scientific) according to the manufacturer’s instructions, and expression levels of the transcription factor and *S100a10* was analyzed by quantitative PCR, 48 h after transfection. siRNA efficciency was calculated based on the downregulation of the transcription factor by its specific siRNA. The experiments were conducted in a 12-well plate with at least three biological replicates. The siRNAs used are *Atf3*, s129666, s129668, *Foxo1*, s136654, s136655, *Lrrfip1*, s173268, s173270, *c-Myc*, s128068, s128069, *Nfkb*, s135615, s135616, *Stat5a* s128672, s128673, *Egr1*, s127689, s127690, *Ets1*, s127719, s127721, *Junb*, s127982, s127983, *Jund*, 201434, 201435, *Fosl1*, s217983, s129817, *Fosl2*, 197390, 197391, *Bhlhe40*, s135199, s135201, *Yy1*, s128676, s128677, *Sp1*, s128429, s128430, *Srf*, s180122, s180123, *Creb1*, s135438, s135439, *Crem*, s130285, s234984, *Stat3*, s129048, s129047, *c-Fos* s66197, s66198, *c-Jun*, s68563, and s201552.

#### Luciferase reporter assays

Assays were performed in N2A cells to identify the functional *S100a10* promoter using the Dual-Luciferase Reporter Assay system (Promega). Exon 1 of *S100a10* with varying lengths of 3′UTR region were cloned upstream of a firefly luciferase reporter construct in a mammalian expression vector pGL4.11 using the SLIC method (Li M 2007). We identified the functional *S100a10* promoter reflected by robust luciferase activity with the coordinates, chromosome3: 93554373–93555181, based on mouse genome assembly, mm10. Next, we identified three *c-Fos* and *c-Jun* binding motifs within this region, and mutated the three sites named mutation1, mutation2, and mutation3 and deleted mutation3. The mutated and deleted firefly luciferase constructs were transfected into confluent Neuro-2a (N2a) cells grown in 12-well culture plates using lipofectamine LTX with Plus reagent (Thermo Fischer Scientific). The renilla luciferase gene was cotransfected as a control for transfection and expression efficiency. Cells were harvested and lysed 48 h after transfection and cell lysates were assayed for firefly and renilla luciferase activity according to the manufacturer’s instructions. Firefly luciferase activity was normalized to renilla luciferase activity for each cell culture well and plotted as activity relative to control transfections. At least four biological replicates were used. For dual transfections, c-Fos and c-Jun siRNA was first transfected using RNAiMAX and then 12 h later, DNA constructs were transfected using lipofectamine LTX with Plus reagent. The cells were harvested 36 h later, when the siRNA effect is maximum. The sequences of all plasmids were verified by sequencing and restriction enzyme digestion.

#### Western blotting

For tissue analysis, mice were anesthetized with CO_2_ and decapitated, and cortex was rapidly dissected, frozen in liquid nitrogen, and stored at −80 °C until further processing. For cell analysis, cells were scraped and collected, rinsed with PBS, flash frozen in liquid nitrogen, and stored at −80 °C. For both tissues and cell pellet, samples were sonicated at 4 °C in lysis buffer containing 20 mM Tris pH 7.5, 150 mM NaCl, 1% SDS supplemented with protease inhibitor (Roche) and phosphatase inhibitor (Roche), and boiled for 10 min. The protein concentration was determined using a BCA protein assay kit (Thermo Fisher Scientific) according to the manufacturer’s instructions. Protein samples were diluted in equal volume of 2× LDS sample buffer (Invitrogen) and supplemented with DTT to a final concentration of 200 mM (Sigma). Twenty micrograms of protein samples were separated on 4–12% Bolt Bis-Tris precast denaturing gels (Invitrogen) and transferred onto PVDF membranes and blocked with 5% milk in TBS–0.1% Tween (TBST) solution for 1 h at room temperature. Membranes were probed with primary antibodies diluted in 5% milk–TBST solution overnight at 4 °C. Membranes were then washed and probed with horseradish-peroxidase-conjugated anti-mouse (Thermo Fischer Scientific, 31460, 1:10,000), anti-rabbit (Thermo Fischer Scientific 31430, 1:10,000), or anti-goat antibody (Jackson Immunoresearch, 305-035-003, 1:10,000) for 1 h at room temperature. Membranes were developed using Pierce Western blotting substrate (Thermo Fischer Sceintific, 32106) and exposed on film. Exposed films were scanned, and protein bands were quantified using ImageJ Software (NIH, USA). Protein quantities were normalized using GAPDH. All values were plotted relative to control/untreated samples. Antisera and antibodies against the following proteins were purchased from the indicated sources: p11 (AF2377, R&D systems, 1:500), GAPDH (Mab374, EMD Millipore 1:1000), total c-Fos (4384, Cell Signaling Technology, 1:300), phospho c-Fos (5348S, Cell Signaling Technology, 1:250), c-Jun (9165S, Cell Signaling Technology, 1:500), phospho c-Jun (3270S, Cell Signaling Technology, 1:500).

#### Chromatin immunoprecipitation

Antibodies against c-Fos (sc-52, Santa Cruz Biotechnology), c-Jun (9165S, Cell Signaling Technology), and rabbit IgG antibody (C15410206, Diagenode) were bound to Protein-G magnetic beads (Diagenode, kch-818-220) for 2 h, at 4 °C (30 μL Protein-G magnetic beads were incubated with 5 μg of Jun antibody, 10 μg of Fos antibody, and 1 μg of IgG antibody. A total of eight frontal cortices from four S100a10-EGFP/Rpl10a ES691 mouse brains from 9-day vehicle- and fluoxetine-treated animals were pooled. They were briefly washed with ice-cold 1× PBS and 1 mM MgCl_2_. Tissue was transferred to a dounce homogenizer and buffer containing 1% formaldehyde, 50 mM HEPES-KOH, pH 7.5, 100 mM NaCl, 1 mM EDTA, 0.5 mM EGTA was added and fixed for 10 min end-to-end rotation. Formaldehyde was then quenched by adding 0.125 mM of glycine for 5 min. Halt-protease and phosphatase inhibiter (Thermo Fischer Scientific and Roche) and chromatin immunoprecipitation (ChIP) cross-link Gold (Diagenode) were added to the buffer before homogenization. Throughout the protocol buffers were supplemented with protease and phosphatase inhibitors. Lysate was then subjected to two wash steps with the same 1× PBS and 1 mM MgCl_2_ at 1350*g* at 4 °C for 5 min. Each sample was washed with lysis buffer 1 containing 50 mM HEPES-KOH, pH 7.5, 140 mM NaCl, 1 mM EDTA, 10% glycerol, 0.2% NP-40, 0.2% Triton X-100 after incubating for 10 min at 4 °C. The pellet was then washed with lysis buffer 2 containing 10 mM Tris-HCl, pH 8.0, 140 mM NaCl, 1 mM EDTA, 0.5 mM EGTA, after incubating at RT for 10 min. The pellet was resuspended in 1 mL shearing buffer at 1 × 10^7^ cells, containing 10 mM Tris-HCl, pH 8.0, 140 mM NaCl, 1 mM EDTA, 0.5 mM EGTA, 0.1% deoxycholate, 0.2% N-lauroylsarcosine, and 0.2% SDS. The samples were sonicated in Covaris settings duty cycle 10% and 175 cycles/burst for 3 min to achieve fragments of sizes ranging from 200 to 500 bp. To each sample, 0.2% Triton X-100 was added and spun at 20,000*g* @ 4 °C for 15 min. The supernatant was collected; 1% of the input sample of each ChIP reaction was collected. The sheared chromatin was immunoprecipitated with washed antibody-bound beads at 4 °C overnight. The antibody coupled beads were washed 2× with low-salt buffer containing 50 mM HEPES-KOH, pH 7.6, 100 mM LiCl, 0.5 mM EDTA, 0.2% NP-40, 0.7% deoxycholate, and then washed 2× with 50 mM HEPES-KOH, pH 7.6, 100 mM LiCl, 0.5 mM EDTA, 0.2% NP-40, 0.7% deoxycholate. Then the beads were washed 2× with 10 mM Tris-HCl, pH 8.0, 50 mM NaCl, 1 mM EDTA, and eluted with 50 mM Tris-HCl, pH 8.0, 10 mM EDTA, 10% SDS buffer and incubated at 65 °C for 1 h. The isolated chromatin and the input samples were immediately cleaned with the IPure kit (Diagenode). The unbound fraction of the immunoprecipitation reaction was used to validate the fragmentation on a 1% agarose gel. Purification with the IPure kit was carried out according to the manufacturer’s instructions with the addition of Proteinase K treatment (Roche) for 1 h at 55 °C after the de-crosslinking step. Immune-enriched chromatin was further purified with phenol:chloroform:isoamyl alcohol (25:24:1) and concentrated by ethanol precipitation. ChIP-sequencing libraries were prepared using the Ovation Ultralow system V2 (Nugen). The samples were further validated, and processed for single reads 75 bp sequencing on the Illumina HiSeq 2500 platform.

#### Genome-wide sequencing

The ChIP-seq library samples were further validated, and processed for single read 75 bp sequencing on the Illumina HiSeq 2500 platform. FastQC was invoked for the sequencing quality control. Good-quality reads were aligned to mouse genome (mm10) with bowtie v1.0.1 (parameter “--best –strata” for keeping only the best hit, default parameters otherwise). Peak regions were called with MACS2 (parameters “-g mm -q 0.01”, Minimum FDR cut-off 0.01 for peak detection, default parameters otherwise) and reads were extended by 200 bp to account for the size of the fragment isolated by the ChIP reaction. Three-way comparisons were performed to identify locations of differential enrichment in the genome between two conditions. This was done by using the tool bdgdiff in MACS2 (parameters “-g 60 -l 120” and “--d1/--d2 according to the callpeak output, default parameters otherwise). IGVTools (IGV Version 2.3.52) were used to convert the pileup peak files into binary tdf files for viewing in IGV. Possible promoter and closest gene of the differential enriched peaks were annotated by R package ChIPpeakAnno; peaks overlapped 5kbp upstream or downstream of a TSS were annotated as possible promoters. We invoke MEME (Version 4.12.0 with parameter -dna -nmotifs 16 -p 8, default parameters otherwise) to discover the motifs with the sequences from the peak regions.

#### RNA purification and quantitative PCR

Mice were anesthetized with CO_2_ and decapitated. The PFC or the whole cortex were rapidly dissected, frozen in liquid nitrogen, and stored at −80 °C. RNA extraction from frozen samples was performed using the Trizol/chloroform technique according to the manufacturer’s instructions (Thermo Fischer Scientific). After extraction, RNA was precipitated overnight at −80 °C in isopropanol with 0.15 M sodium acetate and Glycoblue (Ambion, Austin, TX), washed twice with 80% ethanol, air-dried, and resuspended in nuclease-free water. Purified samples were analyzed using a Nanodrop 1000 spectrophotometer (Thermo Fischer Scientific) in order to assess mRNA quantity and quality.

cDNA was prepared from DNase-treated total RNA using the High Capacity RNA-to-cDNA kit (Thermo Fischer Scientific). Relative gene expression of the cDNA was assayed by qRT-PCR using pre-designed recommended Taqman gene expression assays from Applied Biosystems (ABI) following the manufacturer’s recommendations. Cycle counts for mRNA quantification were normalized to *Gapdh*. Relative expression (ΔCt) and quantification (RQ = 2 –ΔΔC) for each mRNA were calculated using the ΔΔCt method as suggested and the graphs were plotted. Calculation of standard deviation (SDΔCt = (SDtarget2 + SDref2)1/2) and error bars (RQ1 = 2^(−(ΔΔCt+SDΔΔCt)^) and RQ2 = 2^(−(ΔΔCt-SDΔΔCt))^) was performed according to ABI technical literature Part Number 4371095 Rev B. For the control samples, the RQ values are close to 1 but not exactly 1, because the ΔΔCt was obtained after substracting the ΔCt of each control sample from the average ΔCt of the biological replicates.

The following pre-designed TaqMan gene expression assays from Applied Biosystems (ABI) were used. The probe names start with Mm for mouse probes (used to test in mouse brain samples and in primary mixed cortical neurons), and Rn for Rat probes (used to test in rat PC12/TrkB cells). *Creb1*, Mm00501607_m1, Rn00578826_m1; *Crem*, Mm04336053_g1, Rn04338541_m1; *Egr1*, Mm00656724_m1, Rn00561138_m1; *c-Fos*, Mm00487425_m1, Rn00487426_g1; *Fosl2*, Mm00484442_m1, Rn00564121_m1; *Fosl1*, Mm04207958_m1, Rn00564121_m1; *Fosb*, Mm00500401_m1, Rn00500401_m1; *c-Jun*, Mm00495062_s1, Rn99999045_s1; *Junb*, Mm04243546_s1 Rn00572994_s1; *Jund*, Mm04208316_s1, Rn00824678_s1; *S100a10*, Mm00501457_m1, Rn01409218_m1; *Bdnf*, Mm04230607_s1, Rn02531967_s1; *Fgf2*, Mm00433287_m1, Rn00570809_m1; *Egf*, Mm00438696_m1, Rn00563336_m1; *Igf*, Mm00439560_m1, Rn00710306_m1; *Ngf*, Mm00443039_m1, Rn01533872_m1; *Vegf*, Mm00437306_m1, Rn01511602_m1; *Tgfβ*, Mm01178820_m1, Rn00572010_m1; *Gapdh*, Mm_99999915_g1, Rn99999916_s1; *Srsf5*, Mm00833629_g1; *Slc1a2*, Mm01275814_m1; *Sirt1*, Mm01168521_m1; *Glul*, Mm00725701_s1; *Glo1*, Mm00844954_s1; *Crhr1*, Mm00432670_m1; *Adrb1*, Mm00431701_s1; *Abcb1*, Mm00440736_m1.

#### Behavior testing

All behavioral studies were carried out and analyzed with the experimenter blind to the treatment group. Genotypes were decoded after data were processed and analyzed. Procedures were performed as described previously: open-field test (OFT) [40]; novelty suppressed feeding (NSF) [41]; and tail suspension test (TST) [24]. Cohorts of chronic fluoxetine-treated mice were subjected to multiple behavioral testing from a less to worse invasive nature of the tests in the following order: OFT, TST, and NSF.

### Quantification and statistical analysis

Statistical details of each experiment are included in the figure legends. Briefly, for two group comparisons, we used two-tailed unpaired Student’s *t*-test. For multiple group comparisons, we used one-way or two-way ANOVAs and corrections were applied using the appropriate post hoc test. In all experiments, *P* < 0.05 was considered significant. Bar graphs show mean values and the error bars for bar plots are standard error of the mean (±SEM). *N* represents the number of mice for behavior experiments and biological replicates for cell culture experiments. For behavior experiments, all studies were carried out and analyzed with the experimenter blind to the treatment group.

## Results

### AP-1 transcription is stimulated by chronic fluoxetine treatment

**Fig. 1:**
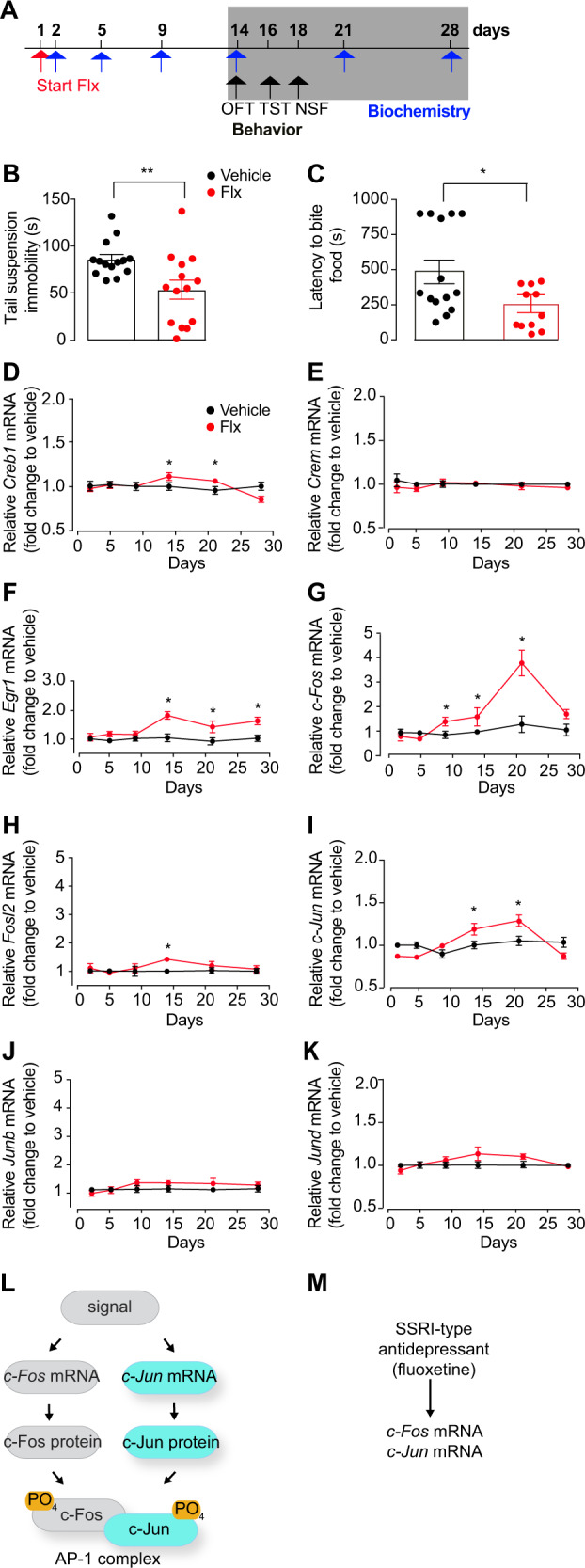
Identification of *c-Fos* and *c-Jun* upregulation during chronic fluoxetine treatment. **a** A schematic diagram showing the timeline for biochemistry and behavior experiments during chronic SSRI fluoxetine (Flx) treatment. Fluoxetine was administered orally to BALB/cJ mice for 28 days. Start of treatment indicated as Day 1 (red arrow). Behavioral analysis (black arrows) using open-field test (OFT) was performed on day 14 (Supplementary Fig. S1), tail suspension test (TST) on day 16, and novelty suppressed feeding test (NSF) on day 18. Biochemistry was done after harvesting the mouse prefrontal cortex (PFC) on 2, 5, 9, 14, 21, and 28 days of treatment (blue arrows). The onset and maintenance of behavioral response is shaded in gray. **b** TST. **c** NSF. **d**–**k** Quantitative-polymerase chain reaction (qPCR) to measure the levels of transcription factor mRNA *Creb1* (**d**), *Crem* (**e**), *Egr1* (**f**), *c-Fos* (**g**), *Fosl2* (**h**), *c-Jun* (**i**), *Junb* (**j**), *Jund* (**k**). **l** A schematic diagram describing the formation of the AP-1 complex. Extracellular signals activate *c-Fos* and *c-Jun mRNA* transcription and protein expression. The proteins get phosphorylated by kinases, forming a stable heterodimeric AP-1 complex, thereby binding to target DNA and controlling their transcription. **m** A schematic diagram depicting *c-Fos* and *c-Jun* regulation by chronic fluoxetine treatment. Statistical analysis was performed between vehicle- (1% saacharine in drinking water) and fluoxetine-treated samples using two-tailed unpaired Student’s *t*-test; *n* = 6 for biochemistry and *n* = 11-14 for behavioral experiments. Data are mean ± SEM; *P ≤ 0.05, **P ≤ 0.01, ***P ≤ 0.005.

To test the hypothesis that IEGs implement specific gene expression programs to mediate an antidepressant response, we aimed to first determine the kinetics of expression of transcription factors in response to treatment with chronic fluoxetine. To do so, we used BALB/cJ mice as they are inherently anxious and produce a robust behavioral response to chronic fluoxetine treatment [22]. We administered fluoxetine orally in drinking water for 28 days to mimic the treatment in humans. The timeline of the behavioral and biochemical experiments is shown in a schematic diagram in Fig. 1a. At various intervals (2, 5, 9, 14, 21, and 28 days of treatment), the PFC was dissected for biochemical analysis and behavioral experiments were performed as indicated.

The response to treatment was assessed by using well-described behavioral paradigms for depressive behavior, including TST and NSF [42]. The fluoxetine-treated mice exhibited reduced immobility in TST (*P* < 0.01, Fig. 1b) and reduced latency to bite food in NSF (*P* < 0.04, Fig. 1c). We observed no effects of the treatment on the locomotor activity of the animals using open-field test (OFT), confirming that the performance of the animals in the TST and NSF tests were not confounded by the overall changes in animal behavior (Supplementary Fig. S1).

Next, to identify transcriptional programs that are initiated in response to antidepressants, we analyzed the gene expression changes of selected families of IEGs in the PFC after days 2, 5, 9, 14, 21, and 28 of fluoxetine treatment. Analysis of the mRNA levels of CREB, Fos, and Jun family IEGs (*Creb1*, *Crem*, *Egr1*, *Fosl1*, *Fosl2*, *c-Fos*, *Fosb*, *c-Jun*, *Junb*, and *Jund)* was performed by quantitative PCR (qPCR) (Fig. 1d–k). Among all the transcription factors tested, *c-Fos* was the most strongly induced gene (Fig. 1g). Surprisingly, *c-Fos* mRNA expression increased to ∼1.5-fold that of the vehicle controls at 9 days of treatment and peaked at ∼3.5-fold at 21 days of treatment. *Fosl1* and *Fosb* mRNA levels were below the threshold for reliable quantification and therefore excluded from analysis. We also observed a statistically significant induction of *Creb1* and *Egr1*, factors that have been previously implicated in neuronal plasticity and neuropsychiatric disorders [11, 12, 43]. As c-Fos is the earliest induced transcription factor in response to fluoxetine and as we wanted to determine the function of molecules that precede initiation of the behavioral response, we decided to focus on c-Fos regulation. In order for c-Fos protein to exert its function, it must be phosphorylated and bound to a member of the Jun protein family, thereby forming a stable functional dimer termed the AP-1 complex (Fig. 1l) [44–47]. The Jun proteins can function either as a homodimer or as a heterodimer by interacting with members of the Fos family. We found that c-Jun is the most likely binding partner of c-Fos based on the coordinated induction of c-Fos and c-Jun between 9 and 21 days of treatment (Fig. 1g, i). In summary, we show that *c-Fos* is the most induced gene among all IEGs in reponse to chronic fluoxetine treatment, and *c-Jun* is the likely binding partner of *c-Fos*, together forming the AP-1 complex (Fig. 1l, m).

### AP-1 controls the expression of neuronal remodeling genes in response to chronic fluoxetine treatment

**Fig. 2:**
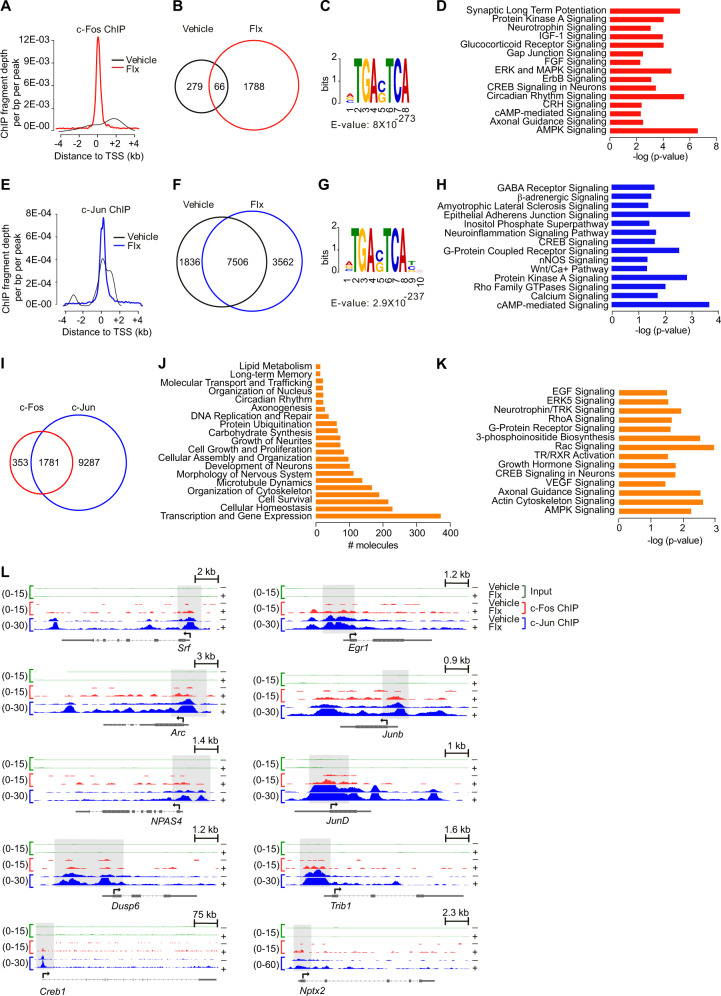
Structural and synaptic plasticity genes are the targets of AP-1 complex in response to chronic fluoxetine treatment. **a**, **e** Aggregate plot of genome-wide ChIP-seq signals for c-Fos and c Jun in the mouse frontal cortex before and after 9 days of Flx treatment at −/+ 4 kb of the transcription start site (TSS). **b**, **f** Venn Diagram showing the overlap in c-Fos and c-Jun binding targets between vehicle- and Flx-treated animals. **c**, **g** Position-weight matrix of the c-Fos and c-Jun binding motif identified using an MEME de novo motif search for all significant peaks genome wide. **d**, **h** Ingenuity pathway analysis (IPA) to identify significant canonical pathways (*y*-axis), which are associated with c-Fos and c-Jun target genes (*x*-axis), displaying the −log *P* value cut-off set to 1.3, calculated by right-tailed Fischer’s exact test. For full gene list, see Supplementary Tables S1 and  S2 and for profiles of depression-associated genes, see Supplementary Fig. S2). **i** Venn Diagram showing the overlap between c-Fos and c-Jun bound target sites. For full gene list, see Supplementary Table S3**. j** Downstream effects analysis examining the genes in the dataset affecting a particular disease or function. **k** Ingenuity pathway analysis (IPA) to identify significant canonical pathways (*y*-axis), associated with c-Fos and c-Jun overlapping sites (*x*-axis), displaying the −log *P* value cut-off set to 1.3, calculated by right-tailed Fischer’s exact test. **l** Genome browser tracks showing the representative profiles of genes bound by both c-Fos (red trace) and c-Jun (blue trace), compared to input samples (green trace), treated with vehicle or fluoxetine (Flx), represented by – and + symbols. A schematic diagram showing the intron–exon structure of each target gene, gene name, and TSS (black arrow to indicate the direction of gene transcription). ChIP peaks near the TSS are shaded in gray. The *y-*axis signal intensity labels are indicated in parenthesis.

The AP-1 complex (Jun–Jun and Jun–Fos dimers) functions as a transcriptional regulator by binding to a common 12-*O*-tetradecanoylphorbhol-12-acetate(TPA)-responsive element (TRE) palindromic sequence, TGAC/GTCA [48]. We hypothesized that the target genes regulated by the Jun–Fos complex may be directly linked to the initiation of the behavioral response to fluoxetine. Hence, we aimed to characterize the targets of the AP-1 transcriptional program using c-Fos and c-Jun genome-wide chromatin immunoprecipitation paired with high-throughput sequencing (ChIP-seq). Mice were treated with fluoxetine for 9 days, the earliest time point at which c-Fos and c-Jun transcription was upregulated (Fig. 1g). Comparison of the target sites between untreated and treated samples from the frontal cortex revealed a number of binding sites in response to drug treatment for both c-Fos and c-Jun (Fig. 2a–g). Analysis of genome-wide binding sites bound by c-Fos and c-Jun at ∓4 kb of the transcription start site (TSS) showed predominant binding to promoter regions of target genes (Fig. 2a, e). In general, we observed c-Fos occupancy on fewer target sites in the vehicle-treated animals reflecting low c-Fos endogenous expression, and c-Fos occupancy on many target sites in response to fluoxetine reflecting robust c-Fos induction (Fig. 2a, b). In contrast, we observed c-Jun occupancy on many target sites in the vehicle-treated mice, indicating high c-Jun endogenous levels, and an even further increase in binding to target sites was observed in response to fluoxetine (Fig. 2e, f).

Next, we identified the significantly enriched motifs in all the c-Fos and c-Jun bound target genes by performing a de novo motif search. We found that the consensus TRE sequence was highly enriched for both c-Fos and c-Jun target sites confirming direct binding of AP-1 to DNA (Fig. 2c, g). We then annotated these target genes and used Ingenuity Pathway Analysis (IPA) to determine association of these genes with specific canonical pathways. We observed that many of the c-Fos target genes regulate pathways including CREB, PKA, neurotrophin signaling, synaptic long-term potentiation, circadian rhythm signaling, and synaptic plasticity (Fig. 2d, Supplementary Table S1). Only the pathways with the most significant −log (*P* value) cut-off of 1.3 are shown. As expected, we found that the c-Jun target genes regulated some of the same pathways as c-Fos target genes, but in addition also bound to genes that regulated structural plasticity, such as Rho, Rac signaling, and actin cytoskeleton signaling (Fig. 2h, Supplementary Table S2). The role of these regulatory proteins in synapse development and plasticity has been well established [49–51].

As c-Jun is known to have broader DNA-binding specificity through homodimer binding or heterodimer formation with other Fos proteins, we analyzed the target genes that showed coincidental binding for both c-Fos and c-Jun. We identified a large overlap between the c-Fos and c-Jun bound genes and identified 1781 commonly regulated binding sites. (Fig. 2i, Supplementary Table S3). Using this list of c-Fos/c-Jun overlapping targets, we carried out downstream effects analysis using IPA to find whether genes in this dataset would affect a particular biological process or disease. Overall we identified pathways affecting neuronal morphology, remodeling, and homeostasis as indicated in Fig. 2j. We also determined the association of these genes with canonical pathways, and, as expected observed a number of commonly regulated pathways (Fig. 2k). These results indicate that c-Fos and c-Jun work together as a complex to function as a transcriptional regulator modulating expression of crucial genes essential for the fluoxetine response.

Next we tested whether any of the human MDD-associated genes based on GWAS studies [52, 53] are targets of c-Fos and c-Jun regulation. Interestingly, we found that both c-Fos and c-Jun are bound to many depression-associated genes described in humans. These include *Srsf5, Srf, Slc1a2, Sirt1, Rab4b, Rab3a, Glul, Abcb1b, Glo1, Crhr1, Creb1, Calm2, Calm1, Bdnf, Adrb1,* and *S100a10* (Supplementary Fig. S2). Among the above depression-associated genes, *Bdnf* and *S100a10* (p11) stand out as they are both essential for mediating the antidepressant response in the cerebral cortex and hippocampus, and play a major role in mood and depressive disorders [54, 55]. These results demonstrate that AP-1 regulated target genes have links to human depression and antidepressant response.

Among the c-Fos and c-Jun targets, transcription factors were the most upregulated group across the various categories (Fig. 2j), and representative ChIP-binding profiles of target genes are shown in Fig. 2l. These include transcription factors *Srf, Egr1, Arc, Junb, Npas4, Jund, Creb1*, and other effector genes*, Dusp6, Trib1*, and *Nptx2* in response to fluoxetine.

Taken together, these findings indicate that the specific induction of AP-1 by antidepressant treatment initiates a transcriptional program that modulates the expression of neuronal plasticity genes which then leads to an antidepressant response. These results prompted us to characterize the pathways that regulate AP-1 transcription and activity.

### Regulation of AP-1 transcription and activity

**Fig. 3:**
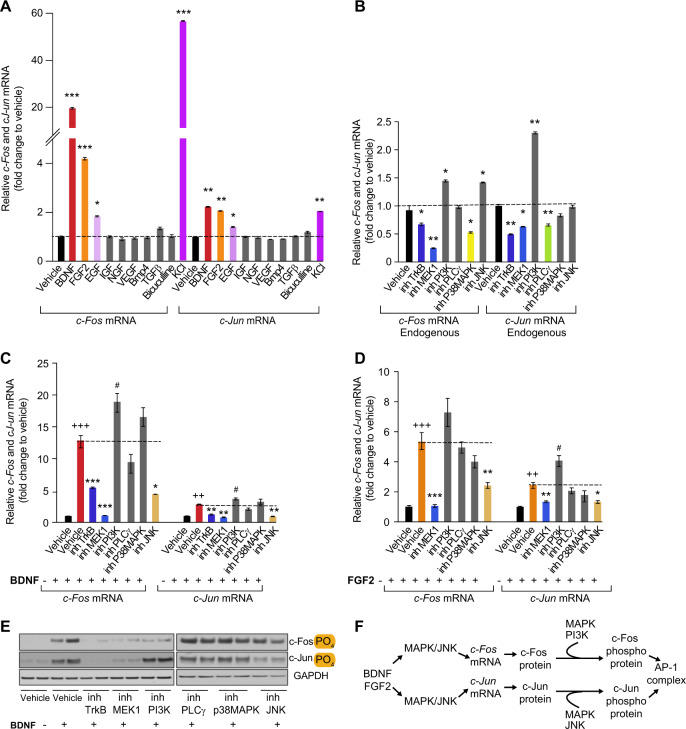
Regulation of *c-Fos* and *c-Jun* mRNA and protein. **a** Stimulation of *c-Fos* and *c-Jun* mRNA in response to acute application of factors, BDNF, FGF2, EGF, IGF, NGF, VEGF, Bmp4, TGFβ, bicuculline, and KCl in DIV 7 primary cortical cultures at 12 h after stimulation by qPCR. **b**–**d** Determination of basal (**b**), BDNF (**c**), or FGF2 (**d**) dependent *c-Fos* and *c-Jun* mRNA expression in response to pharmacological inhibition of the receptor tyrosine kinase pathways in DIV 7 primary cortical cultures by qPCR. Inhibitors for TrkB (K252a), MAPK (U0126, MEK1/2 inhibitor), PI3K (LY294002), PLCγ (U73122), p38 MAPK (SB203850), JNK (SP600125) were used. The figure legends have an “inh” for inhibitor (e.g., inhTrkB for inhibitor of TrkB). **e** Western blots showing the effect of kinase inhibition on BDNF-induced c-Fos and c-Jun phosphorylation. GAPDH loading control blots are shown. **f** A schematic diagram summarizing the BDNF- and FGF2-inducible c-Fos and c-Jun regulation in primary cortical cultures. Statistical analysis was done using one-way ANOVA and corrections for multiple comparisons were performed using post hoc Bonferroni test. Comparisons were made between vehicle- and growth factor-treated samples in **a**, and between growth factor-treated samples with and without inhibitors in **b**–**d**; *n* = 6. In **c** and **d**, + symbol indicates comparison between vehicle- and BDNF- or FGF-treated samples. Data are mean ± SEM; *P ≤ 0.05, **P ≤ 0.01, ***P ≤ 0.005. Dashed lines in **a** and **b** indicate fold change of the control sample, and dashed lines in **c** and **d** indicate fold change of the BDNF- and FGF2-induced sample. The fold change values for the control vehicle samples are normalized to 1.

We show that *c-Fos* and *c-Jun* transcription is stimulated by chronic fluoxetine only after 9 days of chronic treatment. This prompted us to investigate and identify the factors that regulate *c-Fos* and *c-Jun* transcription. The expression of *c-Fos* and *c-Jun* are known to be readily induced by growth factors, neurotransmitters, and electrical stimulation [45, 56, 57]. Therefore, it is possible that growth factor signaling or neuronal activity in response to antidepressant treatment promotes *c-Fos* and *c-Jun* expression. To determine which factors regulate *c-Fos* and *c-Jun* transcription, we established an in vitro primary cortical culture system that allowed for efficient and simultaneous screening of multiple growth factors that could directly stimulate *c-Fos* and *c-Jun* mRNA expression. This cell culture system comprises predominantly cortical neurons and other supporting cell types, and is widely used to study physiological properties of neurons [58]. To capture the peak induction of *c-Fos* and *c-Jun* transcription, we measured their levels 2 h after stimulation (as reported previously [59]) with a variety of growth factors (BDNF, FGF2, EGF, IGF, NGF, VEGF, BMP4, and TGFβ) (Fig. 3a). We also tested neuronal activity modulators such as bicuculline (GABA-A receptor antagonist) and potassium chloride (KCl) that induces depolarization in cultured neurons. *c-Fos* mRNA expression was stimulated by BDNF (∼20-fold), FGF2 (∼4-fold), and EGF (∼1.8-fold) (Fig. 3a). *c-Fos* mRNA expression was significantly stimulated by KCl (∼60-fold), as previously reported [45, 56]. *c-Jun* mRNA expression was similarly induced by KCl, BDNF, FGF2 (all ∼2-fold), and EGF (∼1.2-fold). The other factors showed no effects.

Having demonstrated that BDNF and FGF2 predominantly regulate *c-Fos* and *c-Jun* transcription, we next addressed which specific signaling cascade(s) induces AP-1 transcription and activity. Growth factors act by binding to their cognate receptors and activating MAPK signaling cascades that include extracellular signal-regulated kinases (ERKs), stress-activated protein kinases (JNK/SAPK), and p38 family of kinases (P38 MAPK), all of which stimulate *c-Fos* and *c-Jun* gene expression [60]. Furthermore, the Fos and Jun proteins are functionally regulated via phosphorylation by various kinases. The phosphorylation of c-Fos by ERK and RSK (p90 ribosomal S6 kinase) [61] and the phosphorylation of c-Jun by MAPK and JNK [62, 63] are known to activate their target gene regulation. Using the primary cortical culture system, we screened pharmacological inhibitors of these kinases and assessed the level of *c-Fos* and *c-Jun* transcription in the absence (Fig. 3b) and presence of BDNF (Fig. 3c) and FGF2 (Fig. 3d). Endogenous expression of *c-Fos* and *c-Jun* transcription was strongly attenuated by TrkB (BDNF receptor) and MAPK inhibition, and moderately by p38 MAPK inhibition. No effects were observed upon inhibition of the PLCγ, PI3K, or JNK pathway (Fig. 3c). In the presence of BDNF and FGF2-stimulation, *c-Fos* and *c-Jun* induction was attenuated by TrkB and MAPK kinase inhibition as well as by JNK inhibition (Fig. 3c, d). No effects were observed upon inhibition of other kinases.

To determine which kinases phosphorylate c-Fos and c-Jun proteins, we repeated the inhibitor experiment as above, and tested the levels of phosphorylated c-Fos and c-Jun proteins. Phosphorylation of c-Fos was reduced by MAPK or PI3K inhibition, and c-Jun phosphorylation was reduced by MAPK or JNK inhibition (Fig. 3e). Taken together, these data indicate that BDNF and FGF2 signals regulate both c-Fos and c-Jun transcription via the MAPK and JNK pathways. In addition, the kinases MAPK and PI3K phosphorylate c-Fos protein, and the kinases MAPK and JNK phosphorylate c-Jun protein (Fig. 3f), which leads to the formation of the functional AP-1 complex.

### Specificity of AP-1 transcriptional regulation

To ensure that AP-1 is a specific transcription factor that is relevant for the chronic fluoxetine response, we chose to study in detail the regulation of one of the AP-1 target genes—*S100a10*. *S100a10* expression is downregulated during depressive disorders, is essential for the antidepressant response, and shows enriched expression in specific cell types in brain regions relevant for depression [55]. We also found *S100a10* as a top target gene of AP-1 regulation in our ChIP-seq experiments. Hence characterizing the transcriptional regulation of *S100a10* would further contribute to the understanding of the antidepressant response. We aimed to determine whether AP-1 is the only transcription factor that regulates *S100a10* transcription, or if there is a need for other additional factors which we may have missed during our analysis. To pursue this goal, we first identified the *S100a10* promoter in silico. Based on the ChIP-seq profile, bioinformatic analysis, and search for canonical promoter elements [64], we determined that the exon 1 region of the *S100a10* gene exhibits all the characteristics of a promoter (Supplementary Fig. S3a). Also, our ChIP-seq data revealed that c-Fos and c-Jun bind to this region of the *S100a10* gene. To identify the functional promoter, we cloned varying lengths of the *S100a10* regulatory/promoter region upstream of a luciferase gene reporter, which was used to measure promoter activity (Supplementary Fig. S3b). We transfected these constructs into Neuro-2a (N2a) cells and evaluated the luciferase activity of various constructs. We pinpointed the core functional promoter region of *S100a10*, which is located on chromosome3: 93554373–93555181(based on mouse chromosome assembly, mm10). Next, we searched for a suitable cell line that would enable robust stimulation of AP-1 by BDNF and FGF2, and, in addition, would allow for efficient and targeted transcription factor knockdown following siRNA inhibition of candidate transcription factors.

**Fig. 4:**
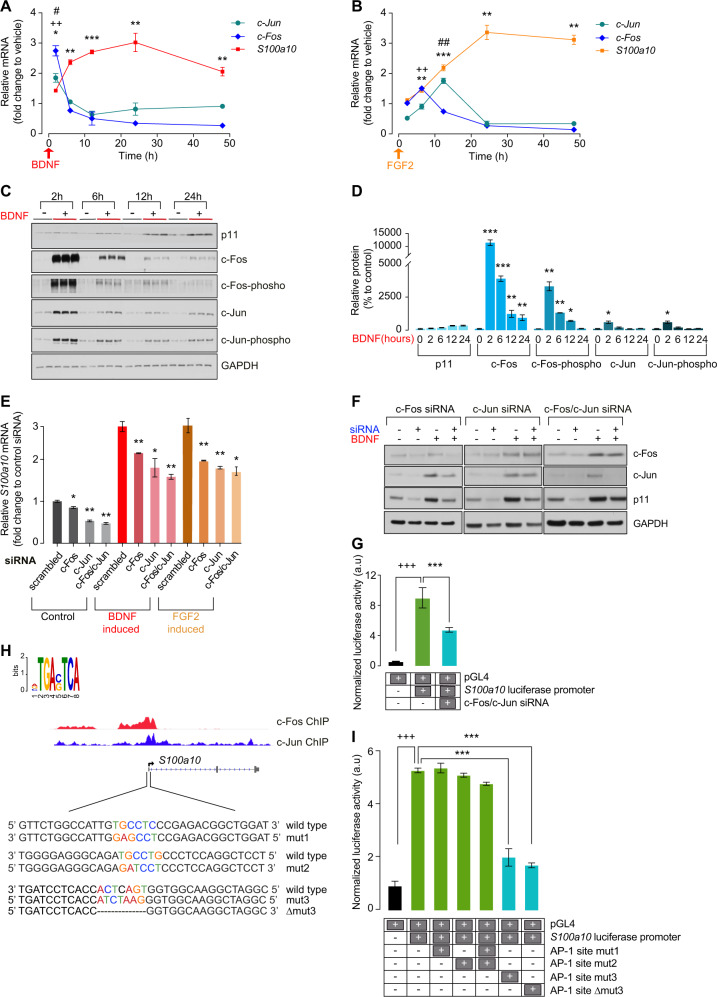
AP-1 specifically regulates the basal and inducible transcription of *S100a10*. **a**, **b** Analysis of *c-Fos*, *c-Jun*, and *S100a10* mRNA expression by qPCR at 2, 6, 12, 24, and 48 h of acute BDNF application (red arrow) and acute FGF2 application (orange arrow) in PC12/TrkB cells. **c** Western blots showing the relative kinetics of p11 (*S100a10*) protein, AP-1 (total and phosphorylated forms of c-Fos and c-Jun), and GAPDH (loading control) after 2, 6, 12, 24, and 48 h of acute BDNF application. **d** Quantification of the blots from **c**. **e** qPCR of basal, BDNF- and FGF2-inducible *S100a10* mRNA expression, when treated with specific siRNAs for *c-Fos*, *c-Jun*, or both. **f** Western blots of c-Fos, c-Jun, and p11 (*S100a10*) protein, untreated (basal) or stimulated with BDNF (induced), and treated with specific siRNAs for *c-Fos*, *c-Jun*, or both. **g** Effect of c-Fos/c-Jun siRNA on *S100a10* promoter activity measured by luciferase reporter gene assay (the predicted *S100a10* promoter sequence is shown in Supplementary Fig. S3a and the identification of the functional promoter is shown in Supplementary Fig. S3b). **h** The consensus AP-1-binding motif is shown on the top. The binding of c-Fos and c-Jun to the promoter region of *S100a10* gene from the ChIP-seq experiment is shown below. The *S100a10* exon 1 promoter sequence comprising potential binding sites for the AP-1 TRE consensus motif were mutated and are denoted as mut1, mut2, and mut3 as shown in the lower panel. The mut3 sequence was deleted and denoted as Δmut3. **i** *S100a10* promoter activity was measured using the luciferase reporter assay to test the effects of AP-1 site mutations in mouse N2A cells. For samples in **a, b**, and **d**, comparisons were made between untreated and treated conditions (*n* = 3) for all samples. Statistical analysis was done using two-way ANOVA to test effects of BDNF or FGF2 treatment and time; corrections for multiple comparisons were performed by running post hoc Tukey’s multiple comparisons test. For **e** and **i**, statistical analysis was done using one-way ANOVA and corrections for multiple comparisons were performed using post hoc Bonferroni test. In **a** and **b**, *P* value significance indicated by symbols # for *c-Jun*, + for *c-Fos*, * for *S100a10*. In **e**, comparisons were made between each scrambled siRNA control and the three siRNA treatments (control, BDNF-, and FGF2-induced). The + symbol represent *t*-test between vehicle- versus BDNF- or FGF2-treated samples. In **i**, comparisons were made between empty pGL4 vector and *S100a10* luciferase promoter construct (+); and between *S100a10* promotor construct and the three AP-1 site mutations (*). In **g**, two-tailed unpaired Student’s *t*-test was performed between the WT *S100a10* promotor construct and the mutant pGL4 constructs (*n* = 4). In **g** and **i**, the + symbol represent *t*-test between vehicle versus BDNF- or FGF2-treated samples. Data are mean ± SEM; *P ≤ 0.05, **P ≤ 0.01, ***P ≤ 0.005.

We identified the rat PC12-TrkB cell line (PC12 cells that stably express the BDNF receptor, TrkB) as a suitable system to study growth factor signaling. These cells demonstrated robust stimulation of AP-1 expression in response to BDNF and FGF2 (Fig. 4a, b). To evaluate the dynamics of expression of these molecules in the PC12-TrkB system, we measured the kinetics of *c-Fos*, *c-Jun*, and *S100a10* mRNA by qPCR. *c-Fos* and *c-Jun* mRNA were rapidly stimulated within 30 min of BDNF or FGF2 application, with a peak induction at 2 h as observed in primary cortical cultures. In contrast, *S100a10* mRNA expression was first observed at 2 h (when c-Fos and c-Jun expression were at their peak), and the highest expression was observed at 24 h after treatment. (Fig. 4a, b). To further validate the expression kinetics, we analyzed their protein levels after BDNF stimulation. The expression dynamics of p11 (*S100a10*) protein and the total and phosphorylated forms of c-Fos and c-Jun were congruent to the mRNA levels, as illustrated in Fig. 4c and quantification of the blots shown in Fig. 4d. We observed the initial induction of *S100a10* mRNA (2 h) at the same time we detected the phosphorylated forms of c-Fos and c-Jun (when they are able to form the AP-1 complex) (Fig. 4c).

Having identified the peak time of induction of *S100a10* at 24 h, we next tested which of the transcription factors, when silenced by specific small-interfering RNAs (siRNAs), affected *S100a10* transcription. We tested 22 candidate transcription factors, all of which have potential to bind to the *S100a10* promoter region. The siRNA inhibition efficiency for each factor and their effect on modulating *S100a10* mRNA expression are indicated in Table 1. Only siRNA against *c-Fos*, *c-Jun*, or both decreased *S100a10* mRNA (Table 1, Fig. 4e, gray bars), with an siRNA efficiency of 55% for *c-Fos* and 67% for *c-Jun*. The siRNA inhibition of *Jund* or *Junb* had no effect. These results further strengthen our previous findings confirming that c-Jun is indeed the authentic binding partner for c-Fos, and that the AP-1 complex specifically regulates *S100a10* transcription.

**Table 1:** Identification of transcription factor(s) regulating p11 by RNAi.

Transcription factor gene symbol	siRNA efficiency	*S100a10* mRNA levels (fold change compared to scrambled siRNA)
*c-Jun*	67%	0.6****
*c-Fos*	55%	0.7*
*c-Fos/c-Jun*	60%	0.5***
*Bhlhe40*	65%	1.2*
*Crem*	63%	1.7*
*Fosl2*	43%	1.3*
*Stat3*	74%	1.5*
*Sp1*	72%	1.5****
*Srf*	41%	1.3*
*Fosl1*	72%	Unchanged
*Junb*	53%	Unchanged
*Jund*	55%	Unchanged
*Atf3*	64%	Unchanged
*Foxo1*	60%	Unchanged
*Lrrfip1*	45%	Unchanged
*Myc*	67%	Unchanged
*Nfkb*	61%	Unchanged
*Stat5a*	63%	Unchanged
*Egr1*	46%	Unchanged
*Cbp/P300*	66%	Unchanged
*Ets1*	68%	Unchanged

Sequence-specific siRNAs against candidate transcription factors were transfected into PC12-TrkB cells and the effect of inhibition of each factor on *S100a10* expression was analyzed after 48 h by qPCR (see Fig. 4e, Supplementary Fig. S4). Comparisons were made between scrambled siRNA control and transcription factor-specific siRNA (*n* = 3). The expression levels of the factors were measured to assess their transfection efficiency. Statistical analysis was done using one-way ANOVA and corrections for multiple comparisons were performed using post hoc Bonferroni test. Data are mean ± SEM; *P ≤ 0.05, **P ≤ 0.01, ***P ≤ 0.005, ****P ≤ 0.0005.

*P value significance; activators of *S100a10* transcription (bold font), repressors of *S100a10* transcription (italic font), unchanged (regular font).

In contrast, siRNA inhibition of factors *Bhlhe40*, *Crem*, *Fosl2*, *Stat3*, *Sp1*, and *Srf* upregulated *S100a10* expression (Table 1, Supplementary Fig. S4a–f), indicating that they are potential repressors.

Next we tested whether both BDNF- and FGF2-dependent induction of *S100a10* requires AP-1 activity. We observed a decrease in BDNF-inducible (red bars in Fig. 4e) and FGF2-inducible (orange bars in Fig. 4e) *S100a10* mRNA when *c-Fos*, *c-Jun*, or both were silenced. To confirm these results, we also measured the protein levels of c-Fos, c-Jun, and p11. We demonstrate that the p11 protein was also downregulated by silencing of c-Fos, c-Jun, or both (Fig. 4f). Our results confirm that AP-1 activity is necessary for both the BDNF- and FGF2-dependent regulation of *S100a10* expression.

Having identified the specific regulation of *S100a10* by AP-1 and determined the potential binding of c-Fos and c-Jun to the *S100a10* promoter region by ChIP-seq, we analyzed if *S100a10* promoter activity would be affected by silencing AP-1 activity using specific siRNAs for both *c-Fos* and *c-Jun* (AP-1 siRNA). The *S100a10* luciferase promoter construct was transfected into mouse Neuro-2a (N2a) cells in the presence and absence of AP-1 siRNA (Fig. 4g). Strikingly, AP-1 depletion resulted in decreased luciferase activity. This indicated that the binding site for AP-1 is indeed located within the identified functional promoter sequence. Hence we searched for the consensus AP-1 motif (TGAC/GTCA) within *S100a10* exon 1 and identified three potential binding sites (Fig. 4h). We then mutated these sites to block AP-1 binding and called them as mutation1 (mut1), mutation2 (mut2), mutation3 (mut3), and deleted mut3 (Δmut3) (Fig. 4h). Mut1 and mut2 contain AP-1-binding motifs oriented in the sense direction, whereas mut3 contains an AP-1-binding motif oriented in the antisense direction. Normalized luciferase reporter activity was measured after transfection of mutants: mut1, mut2, mut3, and Δmut3. Reduced luciferase activity was only observed when the AP-1 consensus motif in mut3 was mutated or deleted (Fig. 4i), confirming that the functional AP-1-binding site is within the *S100a10* promoter sequence. Therefore, our results indicate that the specific binding of AP-1 to the mut3 site within the *S100a10* promoter is likely critical for implementing a robust antidepressant response.

### AP-1 activity is essential for the antidepressant response in vivo

**Fig. 5:**
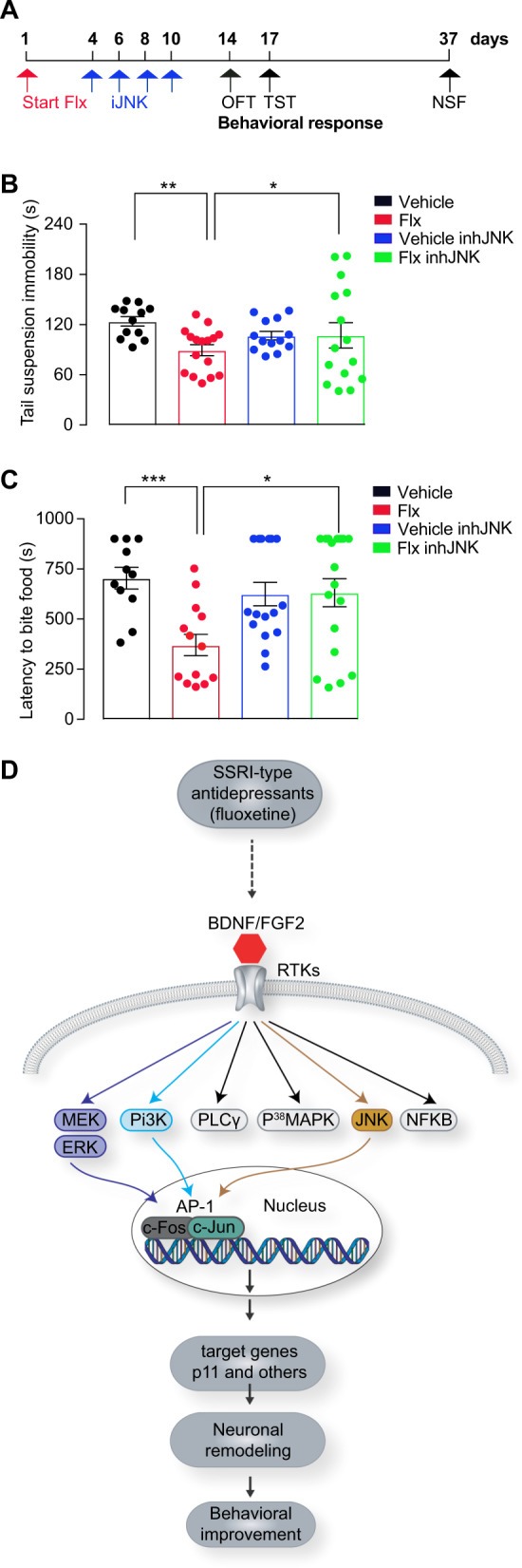
Blocking AP-1 function by inhibiting JNK activity attenuates the antidepressant response. **a** A Schematic diagram showing the timeline of chronic Flx treatment that was initiated on day 1 (red arrow). The JNK inhibitor, SP600125, was injected intraperitoneally (i.p) on days 4, 6, 8, and 10 (blue arrows) of treatment, and behavioral tests (black arrows) of OFT, TST, and NSF were performed on days 14, 17, and 37 of treatment, respectively. **b** Four groups of mice (*n* = 12–18) for vehicle-treated, Flx-treated, vehicle/JNK inhibitor-treated (inhJNK), and Flx/JNK inhibitor-treated were tested for their immobility in TST. **c** Four groups of mice (*n* = 10–18 per group) were subjected to NSF test to measure their latency to bite food in a novel environment. The drinking water consumption and OFT data are shown in Supplementary Fig. S5a and b respectively. A few of the AP-1 target genes affected by inhJNK treatment are shown in Supplementary Fig. S5c. **d** A schematic diagram illustrating the molecular programs and the sequence of signaling pathways that are activated during chronic antidepressant response is shown. The dotted line between fluoxetine and BDNF/FGF2 indicate that SSRIs are known to stimulate BDNF/FGF2 signaling. Statistical comparisons were made using two-way ANOVA to test effects of flx and inhibitor-treatment and were corrected for multiple comparisons by running a post hoc Tukey’s multiple comparisons test. Data are mean ± SEM; *P ≤ 0.05, **P ≤ 0.01, ***P ≤ 0.005.

Finally, we examined whether inhibition of AP-1 function would affect fluoxetine efficacy in vivo. We chose to block JNK as our previous experiments showed that JNK function is essential for the inducible expression of *c-Fos* and *c-Jun* (Fig. 3c, d), whereas MAPK function is necessary for both basal and inducible expression; and JNK selectively phosphorylates c-Jun (Fig. 3e). We used the JNK inhibitor, SP600125, that was previously shown to selectively block JNK 1, 2, 3, and prevent c-Jun phosphorylation when injected into mice intraperitoneally (i.p) at a concentration of 16 mg/kg [65]. We chose to use this concentration of 16 mg/kg rather than 30 or 50 mg/kg [66, 67] to avoid any stress-inducing effects. We used a milder treatment paradigm by injecting the inhibitor on days 4, 6, 8, and 10 of fluoxetine treatment, only when JNK function is likely essential for the antidepressant response (Fig. 5a). We had four groups of animals: Vehicle; Vehicle Fluoxetine; Vehicle inhJNK; Fluoxetine inhJNK; and we powered this study with a large sample size (*n* = 10–18) to overcome influence of variability. We observed that the JNK inhibitor had no effect on the body weight of the mice and the animals drank about 3–6 mL of fluoxetine per day (Supplementary Fig. S5a). The JNK inhibitor would likely inhibit cJun phosphorylation and block the formation of the AP-1 complex. Therefore, the low dose and the mild JNK-inhibitor treatment, while effective at attenuating the onset of the fluoxetine response, did not produce long-term stress-like behavioral effects. We utilized NSF and TST tests to measure the behavioral effects of antidepressant treatment in the four groups of mice as described in Fig. 5a. We observed a robust antidepressant response to fluoxetine reflected by reduced tail suspension immobility (*P* < 0.003) and reduced latency to bite food (*P* < 0.003) in the fluoxetine-treated animals compared with the vehicle controls (Fig. 5b, c). Strikingly, we observed a blunted fluoxetine response in the presence of JNK inhibitor, shown by comparing the fluoxetine-treated group with fluoxetine/JNK inhibitor-treated animals in both the behavioral tests of TST (*P* < 0.02) and NSF (*P* < 0.01). The injection of the JNK inhibitor itself on vehicle-treated animals did not affect their behavior (Fig. 5b, c). We observed no effects of the inhibitor on the locomotor activity of these animals as shown by the open-field test (Supplementary Fig. S5b). In addition, we also determined the expression levels of a representative set of depression-associated AP-1 target genes. We observed that several of the target genes were modulated by fluoxetine and this regulation was partially reversed by the JNK-specific inhibitor treatment (Supplementary Fig. S5c). These data indicate that the JNK inhibitor was effective in blocking target gene expression in the mouse cortex that is relevant for the response. Together, our results demonstrate that the JNK pathway, which we have shown is specifically required for inducing AP-1 activity, is a critical component to activate specific molecules essential for mediating the antidepressant response in vivo.

In summary, our findings reveal activation of a molecular cascade pertaining to *S100a10* regulation during chronic fluoxetine response. The cascade involves the activation of BDNF and FGF2 growth factor signaling, which in turn stimulates MAPK, JNK, and PI3K intracellular pathways to activate an AP-1-driven transcriptional program that regulates neuroplasticity-associated effector genes, which ultimately produce the antidepressant response (Fig. 5d). Altogether, our results highlight the need for temporal activation of select molecules and the significance of sequential signaling events during antidepressant response.

## Discussion

The molecular mechanisms underlying the delayed onset of action of antidepressant drugs is highly debated in the field and is not clearly understood. Both animal and human research has provided supporting evidence that chronic stress and neuropsychiatric disorders have deleterious effects on the brain, both structurally and functionally [5]. Emerging evidence on the role of neuroplasticity and its correlation with behavioral improvement in humans [68] and in mouse models [5, 69] explains the time lag needed to reorganize and remodel the synaptic morphology changes and neural networks disrupted during depression. However, the molecular mechanism is not clearly understood. Here we performed a detailed study to characterize the molecular response to fluoxetine, an SSRI that is widely prescribed for the treatment of several neuropsychiatric disorders. By looking for the early transcription changes during the response, we identified the activation of a selective AP-1 transcriptional program that precedes the onset of the behavioral response in rodents [3, 70], and clinical efficacy in humans [71]. Importantly, a sudden drop in suicidal rate and ideation in humans at 9 days after treatment has been reported [72]. These findings indicate that there is a functional relationship between the genes that are induced at this time point and the behavioral response. Interestingly, we identified effector molecules of the AP-1 transcriptional program that particularly regulate the expression of neuronal remodeling and plasticity-inducing genes, many with known links to depression and antidepressant responses such as *S100a10*. Additionally, mice with a brain-specific deletion of *c-Fos* and *c-Jun* show defects in synaptic plasticity and axonal regeneration respectively [73, 74]. Our findings demonstrate that the onset of the AP-1 transcriptional program links neuronal plasticity to the antidepressant response. However, we cannot rule out the involvement of other transcription factors, microRNAs and RNA-binding proteins that possibly contribute to the observed antidepressant response, as we have mapped the antidepressant pathway by focusing on factors controlling *S100a10* transcription.

The delay in the stimulation of c-Fos and c-Jun transcription is intriguing as AP-1 transcription is shown to be regulated within 30 min of stimulation by growth factors, neurotransmitters, and electrical stimulation [45, 56, 57]. In general, AP-1 function and activity is shown to be regulated by (1) the composition of the Fos:Jun complex (Fos:Jun heterodimer or Jun:Jun homodimer) [45]; (2) by AP-1 binding to transcription factors that regulate gene activation [75] or gene repression [76]; (3) by the ability of AP-1 to affect chromatin accessibility [77]; (4) by AP-1 binding to enhancers [78]. Here we demonstrate that neuronal activity (stimulation by KCl), BDNF and FGF2, two well established growth factors necessary for antidepressant action [54, 79], are the strongest stimulators of *c-Fos* and *c-Jun* transcription in cortical cultures. As sustained neuronal activity is known to induce BDNF-mediated TrkB signaling [80], and as AP-1 is part of the BDNF-positive feedback loop [23], it is likely that BDNF and FGF2 are the rate-limiting factors during the antidepressant response. The molecular steps mediating the interaction between immediate increase in serotonin and BDNF activity during chronic fluoxetine treatment is not completely characterized. However, bidirectional regulation between these two signaling systems and their distinct neuronal functions in survival, neurogenesis, and synaptic plasticity has been well documented [81–84]. Also, *S100a10*-expressing corticostriatal neurons in the cerebral cortex have been shown to exhibit distinct serotonin responses during stress and fluoxetine response indicating the essential role of these neurons in antidepressant action [85]. Moreover, evidence for AP-1 regulation by serotonin signaling [86–89], further substantiates the role of the signaling network comprising serotonin, BDNF, AP-1, and *S100a10* in the initiation of the antidepressant response.

Our studies identifying the role of the MAPK, PI3K, and JNK cascades during BDNF- and FGF2-dependent regulation of the AP-1 complex, together with their reported function in mediating structural and synaptic plasticity function(s) confirm a significant role for these cascades during the antidepressant response. Such neuroadaptive properties have been documented for BDNF [90–92]; FGF2 [93]; MAPK [94–96]; PI3K [97]; JNK [98]; c-Jun [43]; and c-Fos [9]. Interestingly, JNK^*−/−*^ mice have disorganized cortical layers and impaired dendritic architecture due to disrupted microtubule integrity [99]. Consistent with these observations, we have identified Rho, Rac, and cytoskeletal signaling molecules as targets of AP-1 during the fluoxetine response. Although not much is known about the specific function of S100a10 in neuronal plasticity, its role in mediating BDNF-dependent structural plasticity has recently been demonstrated [100]. These results further substantiate and favor the idea that AP-1 regulated genes operate in various molecular and cellular remodeling pathways mediating neuroplasticity.

We establish that AP-1 function is essential for the fluoxetine response. We chose to inhibit the JNK pathway based on key observations. First, our data showed that the JNK pathway was the only pathway that was essential for the inducible expression of *c-Fos* and *c-Jun* in response to BDNF and FGF2-stimulation, whereas the MAPK and PI3K pathways were required for both basal and inducible expression. Also, the role of MAPK in the induction of depressive behavior has been documented before [96]. Second, our experiments showed that JNK is the kinase that predominantly phosphorylates c-Jun, which would then promote DNA-binding and regulation of target effector molecules [63]. Third, our experiments showed that the substrate of JNK, c-Jun, is induced in response to BDNF and FGF2 in vitro and during chronic fluoxetine treatment in vivo. These observations indicate that the JNK function becomes essential under conditions that require *c-Fos* and *c-Jun* induction and Jun phosphorylation, potentially during chronic fluoxetine response. Our results show that an attenuation in the fluoxetine response occurs when the activity of JNK is inhibited, thus validating that AP-1 function is necessary for providing behavioral response to fluoxetine in mice. Although we show evidence that both c-Fos and c-Jun are necessary to regulate the transcription of genes essential for the antidepressant response, we cannot rule out the effects of the AP-1 complex formed by the Jun-Jun homodimer.

We show that the transcriptional regulation of the target genes by the AP-1 complex is selective. By characterizing the regulation of one of the AP-1 target genes, *S100a10*, we demonstrate that the AP-1 complex comprised of c-Fos and c-Jun (and not any of the other members of the Jun-Fos protein family) is the only transcriptional activator, among 22 other transcription factors tested. Furthermore, we identified the AP-1 binding site within the *S100a10* promoter, and proved with nucleotide resolution that this binding site is functionally active, and is likely important for the antidepressant response. In addition, we also identified transcriptional repressors (Bhlhe40, Crem, Fosl2, Stat3, Sp1, and SRF) of *S100a10* gene that may a play a role in the regulation of basal- versus inducible transcription. These findings reveal that the activators and repressors of *S100a10* transcription, together, potentially regulate the cell-type-specific expression of *S100a10* and its signal-dependent induction—both of which are potentially important for the antidepressant response.

In summary, we have identified AP-1 target genes with known links to human MDD based on GWAS data, indicating that these molecules could be used as potential biomarkers for predicting antidepressant responses. We have linked a number of molecules including growth factors, signaling cascades, and a fully integrated gene expression program that function consecutively to activate neuronal remodeling pathways to provide the antidepressant response. Importantly the molecules, FGF2 [101], BDNF [102], c-Fos [103], and p11 [104], are also induced by exercise and enrichment reflecting their function in mediating the homeostatic and neuroplasticity mechanisms in the brain. More evidence for this function comes from the observed upregulation of the molecules, BDNF [105] and p11 [55], following treatment with multiple classes of chemical antidepressants or brain stimulation. Future studies to unravel the mechanisms that trigger the activation of these molecules will therefore help design novel strategies for treating all neurological disorders that benefit from improving neuroplasticity.

## Supplementary information

Figure S1

Figure S2

Figure S3

Figure S4

Figure S5

Table S1

Table S2

Table S3

Description of Table S1, S2, S3
