# Supplementary material for: AP-1 controls the p11-dependent antidepressant response
Source: Mol Psychiatry. 2020 May 21;25(7):1364–81. doi: 10.1038/s41380-020-0767-8 (PMC7303013; doi:10.1038/s41380-020-0767-8)
Supplement: Supplementary file 1 — Figure S1 [file 41380_2020_767_MOESM1_ESM.pdf]

A

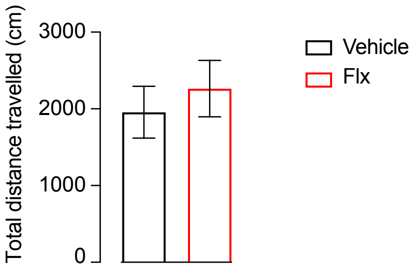

Figure S1. Locomotor function between vehicle- and fluoxetine-treated animals is unaltered.

The vehicle- and fluoxetine (flx)-treated mice were habituated in the testing room in their home cages for 30 min and locomotor activity was assayed for 30 min in an open-field aparatus during 6 consecutive blocks of 5 min. The total distance travelled between the vehicle and the treated mice was measured. We observed no difference between the two groups with respect to their locomotory behavior. Data are represented as mean +/- S.E.M, n=14 mice per group, comparisons made using two-tailed paired t-test.
