# Supplementary material for: AP-1 controls the p11-dependent antidepressant response
Source: Mol Psychiatry. 2020 May 21;25(7):1364–81. doi: 10.1038/s41380-020-0767-8 (PMC7303013; doi:10.1038/s41380-020-0767-8)
Supplement: Supplementary file 2 — Figure S2 [file 41380_2020_767_MOESM2_ESM.pdf]

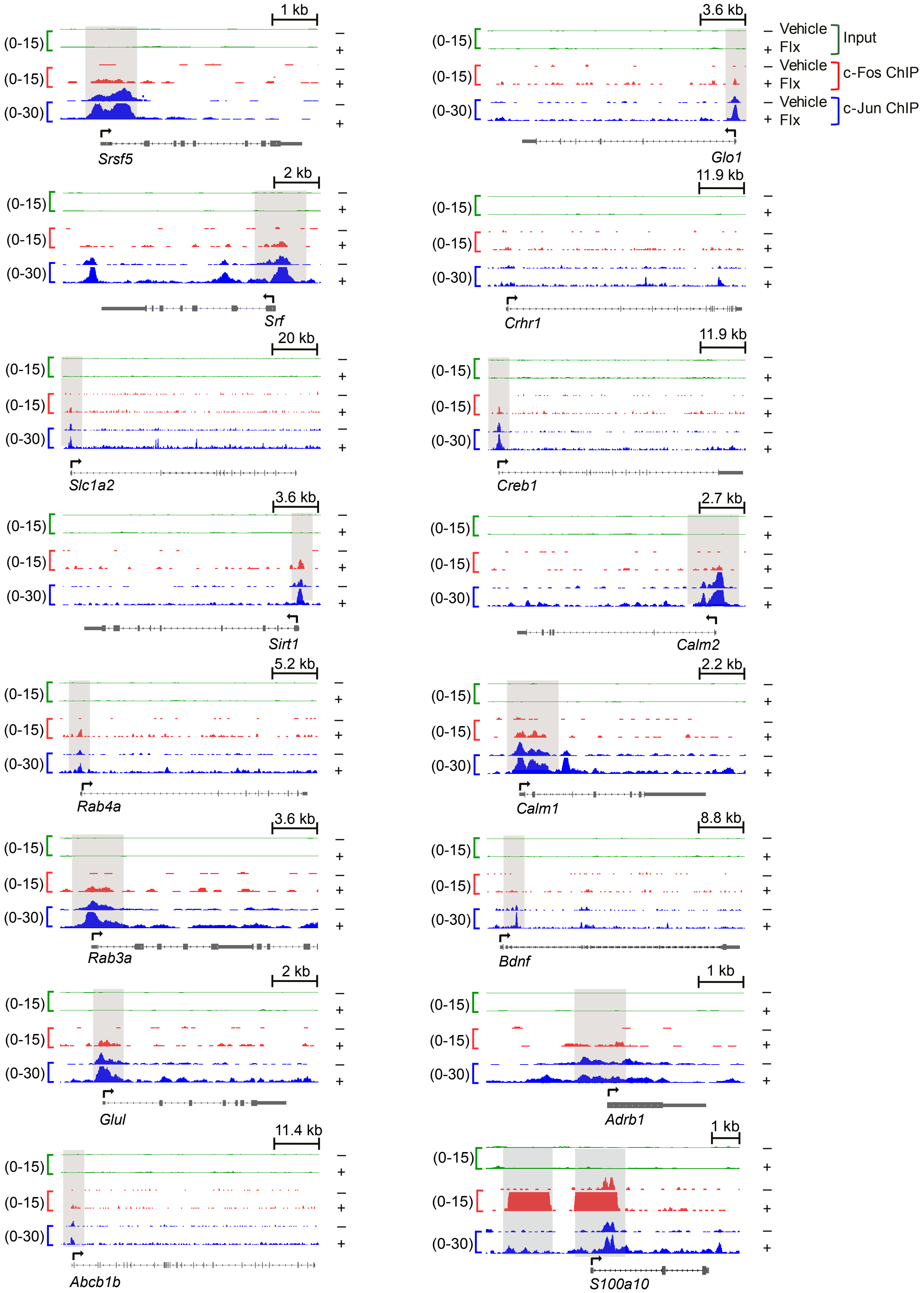

Figure S2. Human depression-associated genes are regulated by AP-1.

Genome browser tracks indicating c-Fos and c-Jun bound to target genes with and without fluoxetine (Flx) indicated by the – and + symbol, inputs (green trace), c-Fos targets (red trace), c-Jun targets (blue trace). The intron-exon structure for each gene is shown below each panel indicating the gene name. Transcription start site is (TSS) indicated by the black arrow representing the direction of gene transcription. The predominant ChIP peaks near TSS are shaded in grey. The Y-axis signal intensity labels are indicated in parenthesis.
