# Supplementary material for: AP-1 controls the p11-dependent antidepressant response
Source: Mol Psychiatry. 2020 May 21;25(7):1364–81. doi: 10.1038/s41380-020-0767-8 (PMC7303013; doi:10.1038/s41380-020-0767-8)
Supplement: Supplementary file 3 — Figure S3 [file 41380_2020_767_MOESM3_ESM.pdf]

A **Predicted mouse *S100a10* exon 1 promoter sequence and promoter elements**

GAGAGAGAGAGAGAGCGCTGGTGGGGCGGGGTGGGGGGCAGGAAGGGAGGGATGGGTGGGGAGGGC
AGATGCCTGCCCTCCAGGCTCCTCCCGCTCCGGGGCGCCTCCGCCCTCTGTACCCGCCTCGCGTACAAA
GACGCGCGGTCTTCGGCACTAGCCTCATCGTGGTGTGCCCAGCTCTTCCAAGGACTGCTGCGCTTCGGG
GCCCAGGTGAGTCCCGCACCTAATATCTGCCCTCTGGGAACCCC

GGGCGCC: BRE Upstream sequence (BREu)
TACAAAGA: TATA-like
GTGGTGTG: BRE Downstream sequence (BREd)
AGTCC: DPE sequence (in the intron)
CCTCATC: Initiator (Inr) sequence

B

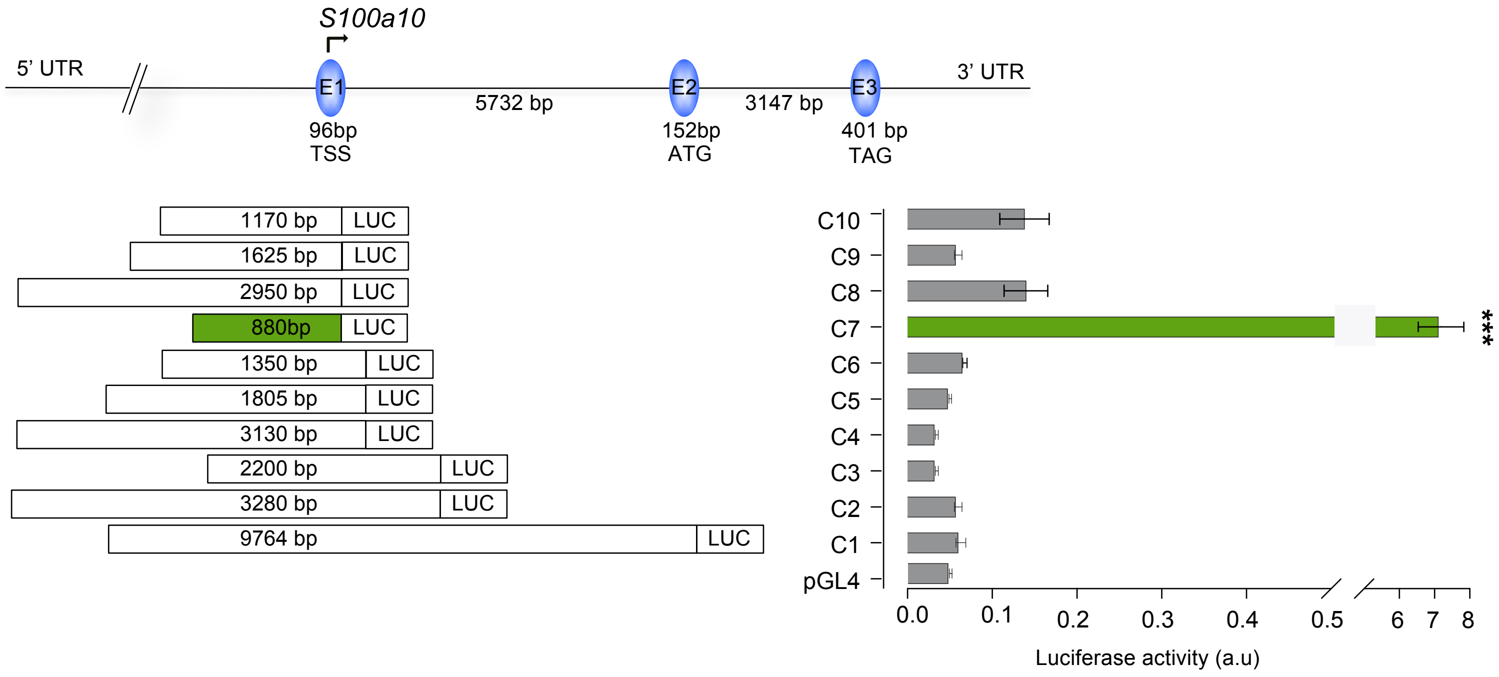

Figure S3. Identification of the *S100a10* promoter.

A. Sequence of the predicted mouse *S100a10* exon 1 (E1) promoter sequence is shown in the top panel. The promoter elements within the sequence are color coded (based on Smale and Kadonaga 2003). **B.** The intron-exon structure of *S100a10* gene comprising exons 1, 2, 3 (E1, E2, E3 are depicted in purple), their respective size, and the 5' and 3' untranslated region (UTR) is indicated. The transcription start site (TSS) is located within exon 1, the translation start site (ATG) is located within exon 2 and the stop codon is located within exon 3 (TAG). The design of the *S100a10* promoter constructs are indicated in the left panel, where different lengths of the *S100a10* promoter regulatory region were cloned upstream of the luciferase reporter gene (LUC) in the pGL4 vector system. Construct 1 (C1) is the longest construct, and the other constructs have varied N-terminal and C-terminal ends as indicated. The last four constructs (C7-C10) stop at the exon-intron junction at the end of exon1. The following three constructs have an additional 180 bp at their C-terminal end. Luciferase activity measurements for all samples are shown in the right panel, corresponding to the constructs on the left panel. We observed robust luciferase activity only for construct 7 (C7), indicating that it is a funtional promoter. Comparisons were made between the control pGL4 vector and the various p11 promoter constructs. Statistical analysis was done using one-way ANOVA and corrections for multiple comparisons were performed using post hoc Bonferroni test. Data are represented as mean +/- S.E.M; *P≤ 0.05, **P≤ 0.01, ***P≤ 0.005, n=4 for each of the constructs.
