# Supplementary material for: AP-1 controls the p11-dependent antidepressant response
Source: Mol Psychiatry. 2020 May 21;25(7):1364–81. doi: 10.1038/s41380-020-0767-8 (PMC7303013; doi:10.1038/s41380-020-0767-8)
Supplement: Supplementary file 4 — Figure S4 [file 41380_2020_767_MOESM4_ESM.pdf]

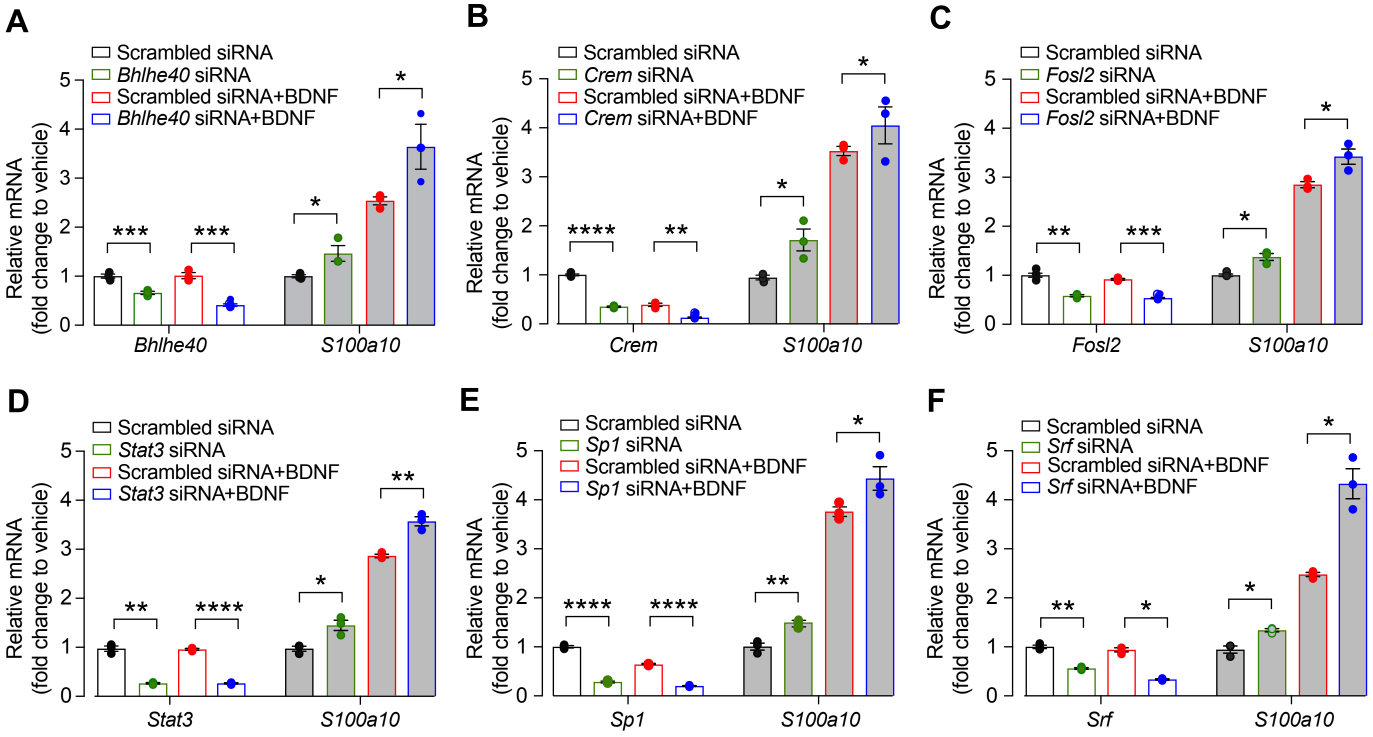


**Figure S4. Transcription factors that repress *S100a10* transcription.**

**A-F**. Sequence-specific siRNAs against candidate transcription factors (**Table 1**) were transfected into PC12-TrkB cells and the effect of inhibition of each factor on *S100a10* upregulation (depicted in red in **Table 1**) is shown. Transfection efficiency was measured by comparing the samples that were treated with scrambled-siRNA control and sequence-specific siRNA for a specific transcription factor (n=3) in the untreated control- or BDNF-treated states (first four bars in each graph). Comparisons were also made to analyze S100a10 transcription between scrambled siRNA control and transcription factor specific siRNAs (n=3), in the basal state or the BDNF-inducible state (last four bars in each graph). For simplicity the comparisons for the differences in expression between control- and BDNF-induced samples are not indicated. Statistical analysis was done using one-way ANOVA and corrections for multiple comparisons were performed using post hoc Bonferroni test. Data are mean +/- SEM; *$P \leq 0.05$, **$P \leq 0.01$, ***$P \leq 0.005$, ****$P \leq 0.0005$.
