# Supplementary material for: AP-1 controls the p11-dependent antidepressant response
Source: Mol Psychiatry. 2020 May 21;25(7):1364–81. doi: 10.1038/s41380-020-0767-8 (PMC7303013; doi:10.1038/s41380-020-0767-8)
Supplement: Supplementary file 5 — Figure S5 [file 41380_2020_767_MOESM5_ESM.pdf]

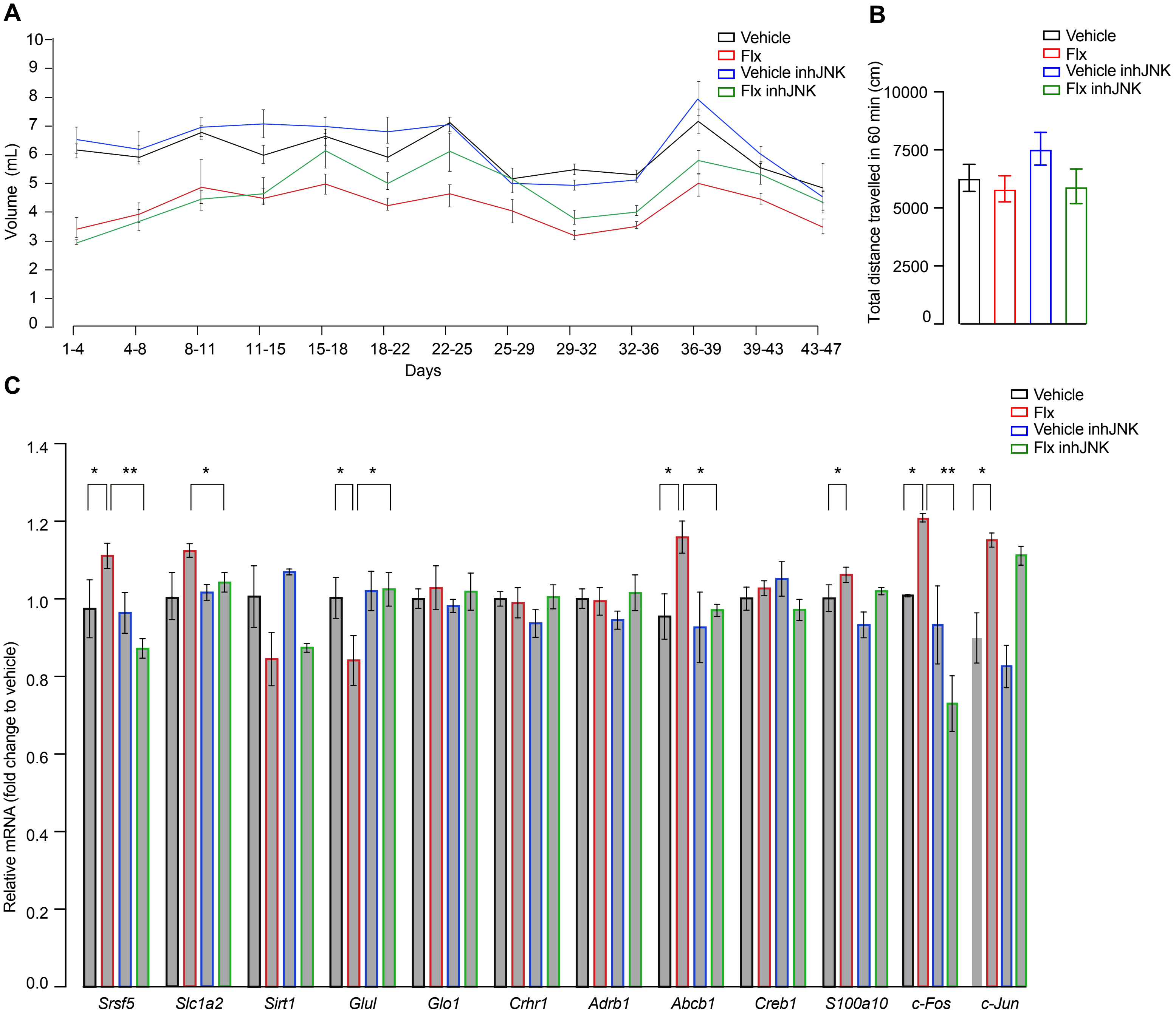

Figure S5. Effects of JNK-specific inhibitor on the fluoxetine response in mice.

A. Volume of liquid intake per day per animal was measured and the average drinking data per group is plotted for the four groups of animals (vehicle-treated animals received 1% saccharine in drinking water to mask the taste of Flx, Flx-treated animals were given 0.167 mg/ml in 1% saccharine). **B.** Open Field data. Animals were habituated in the testing room in their home cages for 60 min and locomotor activity was assayed for 60 min in an open-field aparatus. The total distance travelled between the vehicle and the treated mice was measured. We observed no difference between the two groups with respect to their locomotory behavior. Data are represented as mean +/- S.E.M, n=10-18 mice per group, comparisons were made using two-tailed paired t-test. **C.** qPCR of representative depression-associated AP-1 target genes from the mouse cortex when treated with vehicle, Flx, Vehicle inhJNK and Flx inhJNK conditions. Data are represented as mean +/- S.E.M, n=4 mice per group. Statistical comparisons were made using two-way ANOVA to test effects of fluoxetine- and inhJNK-treatment and were corrected for multiple comparisons by running a post-hoc Tukey's multiple comparisons test. Data are mean +/- SEM; *P≤ 0.05, **P≤ 0.01, ***P≤ 0.005.
