# Supplementary material for: AP-1 controls the p11-dependent antidepressant response
Source: Mol Psychiatry. 2020 May 21;25(7):1364–81. doi: 10.1038/s41380-020-0767-8 (PMC7303013; doi:10.1038/s41380-020-0767-8)
Supplement: Supplementary file 9 — Description of Table S1, S2, S3 [file 41380_2020_767_MOESM9_ESM.docx]

**ADDITIONAL SUPPLEMENTAL INFORMATION**

**Description for Table S1, S2, S3**

**Table S1.** c-Fos ChIP-seq data showing the list of all target sites bound by c-Fos. The file includes three different sheets listing vehicle-only targets, fluoxetine-only targets and common to both conditions.

**Table S2.** c-Jun ChIP-seq data showing the list of all target sites bound by c-Fos. The file includes three different sheets listing vehicle-only targets, fluoxetine-only targets and common to both conditions.

**Table S3**. The file includes overlapping target sites bound by c-Fos and c-Jun.
